# Racial Inequities Influencing Admission, Disposition and Hospital Outcomes for Sickle Cell Anemia Patients: Insights from the National Inpatient Sample Database

**DOI:** 10.3390/hematolrep17030027

**Published:** 2025-05-09

**Authors:** Jayalekshmi Jayakumar, Nikhil Vojjala, Manasa Ginjupalli, Fiqe Khan, Meher Ayyazuddin, Davin Turku, Kalaivani Babu, Srinishant Rajarajan, Charmi Bhanushali, Tijin Ann Mathew, Poornima Ramadas, Geeta Krishnamoorty

**Affiliations:** 1 The Brooklyn Hospital Centre, Brooklyn, NY 11201, USA; mginjupalli@tbh.org (M.G.); fkhan1@tbh.org (F.K.); dturku@tbh.org (D.T.); 2 Trinity Health Oakland, Detroit, MI 48341, USA; nikhil.vojjala@trinity-health.org (N.V.); geeta.krishnamoorthy@trinity-health.org (G.K.); 3 CMH Lahore Medical College and Institute of Dentistry, Lahore 54810, Punjab, Pakistan; meherayyaz@gmail.com; 4 Allegheny General Hospital, Pittsburgh, PA 15212, USA; kalaivani.babu@ahn.org (K.B.); srinishant.rajarajan@ahn.org (S.R.); 5 Saint Vincent Hospital, Worcester, MA 01608, USA; charmi.bhanushali@stvincenthospital.com; 6 Southeast Health, Dothan, AL 36301, USA; tamathew@southeasthealth.org; 7 LSU Health Shreveport, Feist-Weiller Cancer Center, Shreveport, LA 71103, USA; poornima.ramadas@lsuhs.edu

**Keywords:** sickle cell, racial sickle cell, racial inequities, sickle cell anemia

## Abstract

**Background:** Sickle cell disease (SCD) significantly impacts diverse racial groups, particularly African American and Hispanic persons, who experience notable disparities in healthcare outcomes. Despite the extensive literature on SCD, studies focusing on in-hospital racial inequities remain limited. **Methods:** We conducted a retrospective analysis using the National Inpatient Sample (NIS) from 2016 to 2020, identifying adult hospitalizations for SCD (HbSS genotype). Hospitalizations were categorized by race—White, African American, Hispanic, and other, and analyzed for demographic variables, admission types, disposition outcomes, and complications. Statistical analyses included chi-square tests and multivariate logistic regression, adjusting for confounders. **Results:** Of the 1,089,270 identified hospitalizations, 90.31% were African American. African American and Hispanic patients exhibited significantly higher non-elective admissions compared to Whites (77.81%). In-hospital mortality was highest among Hispanics (0.82%). Multivariate regression analysis revealed that African Americans and others had higher odds of prolonged hospital stays (Adjusted Odds Ratio (AOR): 1.30 and 1.20, respectively). African Americans and Hispanics also had increased risks of in-hospital complications of SCD. **Conclusions:** This study highlights substantial racial disparities in SCD hospitalizations, with African Americans and Hispanics facing poorer outcomes compared to Whites. Hispanics also demonstrated increased mortality. These findings underscore the need for targeted healthcare interventions to address racial inequities in SCD management and improve outcomes for all affected populations.

## 1. Introduction

Sickle cell disease (SCD) is an autosomal-recessive hemoglobinopathy which is caused by the replacement of negatively charged glutamine with the neutral valine at the 6th codon of the beta globin chain of Hemoglobin [1]. Historically, SCD is more prevalent in sub-Saharan Africa; however, recent data show that about 20 million people are affected by SCD worldwide and approximately 100,000 Americans have SCD [2,3]. Among African American births in the United States, the frequency of SCD is 1 in 360 live births [4]. The Centers for Disease Control and Prevention (CDC) estimates that 1 in 13 babies born to African American parents have the sickle cell trait, and 1 in 365 African Americans have SCD. Numerous studies underscore the criticality of risk stratification and the inclusion of race in elucidating disease processes. When it comes to SCD, the literature is scarce, and studies have mostly been conducted on targeted populations. Existing research focused on genetic and economic disparities among SCD patients, and studies specifically addressing in-hospital populations are rare [5,6]. Moreover, since most of the affected populations are of African American origin, existing research and therapy is focused on this subgroup which may jeopardize the outcomes of other minority populations with SCD. In this study, we analyzed the impact of race on in-hospital outcomes of SCD patients (HbSS genotype) with a focus on minority races. The study also analyzes the impact of race on admission and disposition outcomes which are known to cause a significant impact on healthcare burden and patient satisfaction [7].

## 2. Methods

### 2.1. Database—The National Inpatient Sample 2016–2020

The National Inpatient Sample (NIS) stands as the United States’ most extensive publicly accessible database for inpatient healthcare, encompassing data from various payers. It serves as a crucial tool for extensive data analysis, offering regional and national insights into inpatient usage, accessibility, costs, quality of care, insurance, demographic information, and clinical outcomes. Created through a collaborative effort between federal, state, and industry partners under the Agency for Healthcare Research and Quality’s Healthcare Cost and Utilization Project (HCUP), it includes information from approximately 20% of hospital admissions every year. As per our institution’s (Trinity Health Oakland) policy, Institutional Review Broad (IRB) approval is not required for studies utilizing deidentified database data; therefore, the use of NIS data for this study was deemed exempt.

### 2.2. Study Population and Study Variables

The NIS 2016–2020 was queried and International Classification of Disease—Tenth Edition—Clinical Modification (ICD-10-CM) codes were used to identify adult (age > 18 years) hospitalizations with a primary or secondary diagnosis of SCD with the HbSS genotype. This population was stratified by race into Whites, African Americans, Hispanics, and others (Asian, Native American, and Pacific Islander). Sociodemographic variables including age, sex, insurance, income quartiles, and hospital characteristics, including hospital size, location, region, and teaching status, were compared across different races. The main outcomes studied were the type of admission (elective vs. non-elective), the type of disposition (home vs. assisted living facility/home healthcare vs. died), and healthcare utilization (a length of stay greater than or less than 7 days). Complications of SCD hospitalizations like acute kidney injury (AKI), pain crisis, deep vein thrombosis (DVT), acute chest syndrome, cerebrovascular accident (CVA), and pulmonary hypertension (PHTN) were also compared across different races.

### 2.3. Statistical Analysis

A cross-sectional analysis was conducted using the STATA/MP 17.0 software. Categorical variables were compared using a chi-square test and continuous variables were compared using a *t*-test. *p* values < 0.05 were considered statistically significant. Multivariate logistic regression analysis was conducted to analyze the impact of race on the type of admission, the type of disposition, healthcare utilization, and complications like AKI, pain crisis, DVT, acute chest syndrome, CVA, and PHTN while accounting for pertinent confounders. These included socio-demographics, hospital characteristics, the Charlson comorbidity index [8], and other cardiac risk factors, including tobacco use, hypertension, hyperlipidemia, diabetes, cannabis use, and obesity.

## 3. Results

### 3.1. Patient- and Hospital-Level Characteristics

A total of 1,089,270 SCD hospitalizations were identified with a mean age of 35.81 years. In total, 65.29% of these patients were women. The majority of SCD hospitalizations were African American (90.31%), followed by Hispanics (4.08%), others (2.99%), and Whites (2.08%).

Less than 20% of hospitalizations among African Americans, Hispanics, and others were older than 65 years. However, among Whites, 36.22% were found to be greater than 65 years (*p* < 0.001). Irrespective of race, the majority of SCD hospitalizations were women (*p* < 0.001). In all race categories, the highest proportion of people had a median household income in the lowest income quartile, with 51.74% of African Americans and 45.91% of Hispanics in this category compared to 31.58% of Whites. Only 9.75% of African Americans SCD patients had income in the highest income quartile compared to 20.54% of Whites (*p* < 0.001). The majority of White SCD patients had private insurance (33.14%) whereas most African Americans (44.75%), Hispanics (53.21%), and others (45.74%) had Medicaid. (*p* < 0.001) Among Whites and African Americans, the majority were hospitalized in the Southern region of the United States whereas Hispanics and others were mostly hospitalized in the Northeast (*p* < 0.001). Irrespective of race, the majority were treated at urban teaching hospitals. (*p* < 0.001) (Table 1).

### 3.2. Primary Outcomes

In all races, most of the population had a routine discharge to home. Whites had a higher percentage of discharge to facility/home healthcare (21.09%) compared to African Americans (11.24%), Hispanics (9.97%), and others (12.3%) (*p* < 0.001). On univariate analysis without adjusting for confounders, Hispanics with SCD had the highest in-hospital mortality (0.82%), followed by Whites (0.8%), African Americans (0.64%), and others (0.54%). (*p* < 0.001). The highest proportion of hospitalizations from all races were admitted non-electively (*p* < 0.001) and had a length of stay less than 1 week (*p* < 0.001) (Table 2).

Multivariate logistic regression analysis, adjusted for confounders was performed after assigning Whites as the reference group. It showed that, compared to Whites, all other race categories had statistically significantly lower odds of being admitted electively to hospitals. It was also found that, as opposed to Whites, African Americans and others had statistically significantly higher odds of a prolonged hospital stay greater than 7 days [Adjusted Odds Ratio (AOR): 1.3 (95% Confidence Interval (CI): 1.17–1.42, *p* < 0.001) in African Americans and AOR: 1.2 (CI: 1.05–1.37, *p* = 0.006) in others]. No statistically significant associations were found between the races in terms of disposition to home vs. facility/home healthcare vs. died (Table 3).

### 3.3. In-Hospital Complications

**Figure 1:**
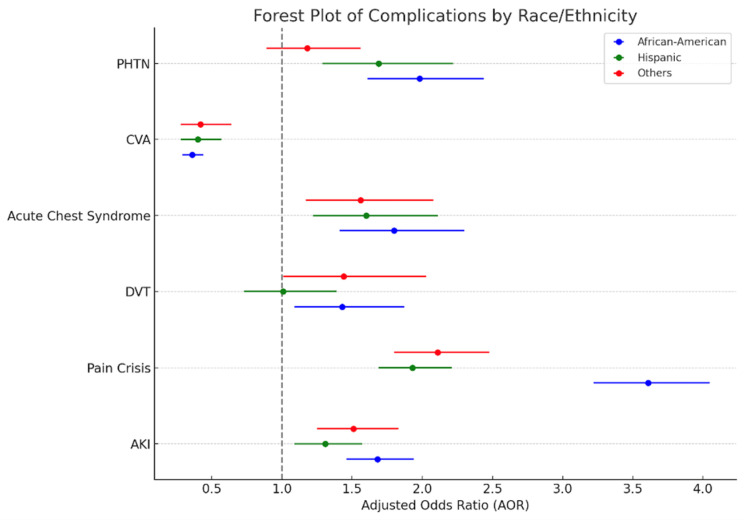
Forest plot showing racial differences in the occurrence of in-hospital complications of SCD patients in the NIS (2016–2020).

Racial differences in the occurrence of the most common complications encountered in the in-patient population of SCD were analyzed using multivariate logistic regression analysis. This showed that, compared to Whites, African Americans, Hispanics, and others had statistically significantly higher odds of developing AKI, pain crisis, acute chest syndrome, and PHTN. On the contrary, the odds of the occurrence of cerebrovascular accidents were found to be lower in all other race categories compared to Whites. Higher odds of DVT were found among African Americans and others compared to Whites; however, the difference in the odds of developing DVT was not statistically significant in Hispanics compared to Whites (Table 4 and Figure 1).

## 4. Discussion

The findings of this study illuminate critical racial disparities affecting SCD patients in the United States during hospital admissions from 2016 to 2020. The distribution and characterization of SCD hospitalizations varied remarkably among races. In addition to having the highest proportion/burden of SCD hospitalizations, African Americans were also found to have significantly poor in-hospital outcomes compared to Whites. Previous studies had smaller sample sizes with more focus on the comparison of African Americans with Whites [9]. Our study also focused on Hispanics, the race category with the second highest prevalence of SCD in the United States (4.8%) [9], along with admission and disposition outcomes and in-hospital complications. Hispanics were also found to have notably worse in-hospital outcomes compared to Whites. This raises important questions about the systemic factors contributing to these inequities.

The analysis of the data reveals a significant disparity in the age distribution of individuals with SCD among different racial groups. Specifically, White patients exhibited a higher proportion of patients >65 years of age compared to the African American, Hispanic, and other racial categories, while the median life expectancy of SCD patients is 42 years for females and 38 years for males [10]. This higher life expectancy in White SCD patients may be due to a higher proportion of Whites in the highest income quartiles and a similar higher prevalence of private insurance among White individuals. This is supported by observations from previous research that have highlighted that SCD patients reliant on Medicare and Medicaid tend to experience lower life expectancies [11]. Additionally, it has been documented that Medicare and Medicaid beneficiaries often face limitations in their choices of nursing homes, with many options being of lower quality, leading to a higher likelihood of rejection compared to the options available to those with private insurance [12]. This may contribute to the observed pattern of higher rates of discharge to nursing homes for White patients. In contrast, other racial groups are more frequently discharged to home settings.

Compared to Whites, African Americans and Hispanics had higher odds of being admitted non-electively which contributes to higher healthcare utilization compared to elective admissions. A previous study has reported a declining trend of non-elective admissions for Whites and an increasing trend of the same for African Americans [13]. The observed a higher length of stay in African Americans can be attributed to a complex interaction between patient and healthcare-related factors like medication adherence, failure of physicians to timely recognize impending complications, difficulty with discharge planning, and suboptimal pain management [14]. The increased length of stay for African American patients could be supported by studies showing increased average wait times in emergency rooms for African American SCD patients leading to delays in care delivery [14]. A recent study also showed that patients who perceived discrimination in their care reported a higher pain burden, which could delay discharge due to an inability to achieve pain score goals [15].

African Americans, Hispanics, and others had higher odds of AKI, pain crisis, and acute chest syndrome compared to Whites. However, higher odds of developing PHTN were found only for African American and Hispanic SCD patients. This is consistent with studies showing a higher prevalence of PHTN in Hispanics and a lower incidence in Asian Americans owing to a lower left ventricular mass [16]. Compared to Whites, all other racial groups had lower odds of developing cerebrovascular accidents due to unclear reasons. An emerging body of research highlights the immune-modulating properties of young red blood cells (RBCs) (such as CD71+RBCs), which may influence complications or inflammatory responses in SCD patients. This offers insight into possible biological mechanisms underlying these disparities [17]. The socioeconomic disparities observed in African Americans in our study might also contribute to worse in-hospital complications among African Americans. While African Americans are well known to have higher disease severity in SCD due to genetic reasons, our study highlights many socioeconomic disparities involving African Americans, Hispanics, and others suggesting that systemic and socioeconomic factors also play a crucial role in driving racial disparities in healthcare.

The NIS lacks detailed patient-level data such as medications and laboratory results which could interfere with the studied outcomes. Thus, it was not feasible to eliminate these as potential confounders for the outcomes studied. The reliance on administrative data from the NIS may introduce potential inaccuracies or coding errors, which could affect the accuracy of diagnoses and identification of complications. As a cross-sectional analysis, this study can only establish associations, not causal relationships, between race and the outcomes examined. Despite efforts to control for confounding variables, there may be unmeasured or residual confounders that were not fully accounted for in the analysis. Since the NIS database only includes data from hospitalized patients in the United States, it may not fully represent the entire world population of SCD, which is more prevalent in Sub-Saharan Africa. As a result, applying our findings to a larger population could pose challenges. The cross-sectional nature of our study limits its ability to assess the frequency and impact of readmissions on the studied outcomes. However, despite these shortcomings, the compelling disparities encountered in our study provoke important inquiries into the impact on race on outcomes of SCD hospitalizations.

As we analyze these results, it is vital to consider the broader implications for healthcare delivery and policy aimed at improving the quality of care for marginalized racial groups. Understanding such health outcome variations among racial groups is crucial for developing tailored diagnostic and management protocols, ensuring equitable treatment efficacy.

## 5. Conclusions

In conclusion, this study reveals significant racial disparities in hospitalization outcomes for SCD patients, particularly highlighting the challenges faced by African American and Hispanic populations. The findings demonstrate that these groups experience higher rates of non-elective admissions, prolonged hospital stays, and increased complications, underscoring the need for targeted healthcare interventions. Addressing these inequities is crucial for improving patient outcomes and ensuring equitable care for all individuals affected by SCD.

## Figures and Tables

**Table 1:** Patient- and hospital-level characteristics of hospitalized adult SCD patients stratified by race in the NIS (2016–2020).

Variables	White [22,710] (%)	African American [981,759] (%)	Hispanic [52,176] (%)	Others [32,569] (%)	*p*-Value
Age					
Less than 65 years	63.78	82.93	87.72	85.06	<0.001
Greater than 65 years	36.22	17.07	12.28	14.94	
Gender					
Male	33.02	35.15	29.18	30.85	<0.001
Female	66.98	64.85	70.82	69.15	
Median household income national quartiles					
Quartile 1 (0–25th percentile)	31.58	51.74	45.91	37.91	<0.001
Quartile 2 (26–50th percentile)	24.37	22.41	23.45	20.84	
Quartile 3 (51–75th percentile)	23.51	16.1	18.7	20.79	
Quartile 4 (76–100th percentile)	20.54	9.75	11.94	20.46	
Insurance Type					
Medicare	31.24	29.11	17.3	16.98	<0.001
Medicaid	28.75	44.75	53.21	45.74	
Private	33.14	19.88	21.66	29.5	
Other	6.88	6.27	7.83	7.77	
Hospital Region					
Northeast	23.15	18.54	45.73	45.36	<0.001
Midwest	18.34	19.92	4.8	9.02	
South	42.63	53.39	38.34	36.05	
West	15.89	8.15	11.12	9.17	
Hospital Location					
Rural	3.8	3.67	1.13	2.33	<0.001
Urban non-teaching	19.93	14.04	12.33	11.49	
Urban teaching	76.27	82.29	86.55	85.17	
Hospital bed size					
Small	20.04	16.35	16.14	14.27	0.1864
Medium	25.37	26.75	27.49	26.84	
Large	54.58	56.89	56.37	58.89	

**Table 2:** Racial differences in admission and disposition outcomes and healthcare utilization among SCD patients in the NIS (2016–2020).

Outcomes	White [22,710] (%)	African American [981,759] (%)	Hispanic [52,176] (%)	Others [32,569] (%)	*p*-Value
Patient disposition					
Routine	78.11	88.11	89.21	87.16	<0.001
Transfer to facility/home healthcare	21.09	11.24	9.97	12.3	
Died	0.8	0.64	0.82	0.54	
Admission Type					
Non-Elective	77.81	87.7	83.3	81.74	<0.001
Elective	22.19	12.3	16.7	18.26	
Length of stay					
Less than 7 days	80.13	78.09	81.16	78.8	<0.001
More than 7 days	19.87	21.91	18.84	21.2	

**Table 3:** Multivariate regression analysis of racial differences in admission and disposition outcomes, and the healthcare utilization of SCD patients in the NIS (2016–2020).

	Elective vs. Non-Elective Admission AOR * [95% CI, *p*-Value]	Facility/Home Health vs. Routine AOR * [95% CI, *p*-Value]	Died vs. Routine AOR * [95% CI, *p*-Value]	Length of Stay > 7 Days AOR * [95% CI, *p*-Value]
White [22,710]	Reference	Reference	Reference	Reference
African American [981,759]	0.5 (0.45–0.56, <0.001)	0.88 (0.69–1.14, 0.352)	1.53 (0.61–3.85, 0.362)	1.3 (1.17–1.42, <0.001)
Hispanic [52,176]	0.75 (0.65–0.84, <0.001)	0.79 (0.57–1.09, 0.159)	2.29 (0.71–7.37, 0.165)	1.06 (0.94–1.2, 0.289)
Other [32,569]	0.8 (0.69–0.93, 0.003)	0.86 (0.61–1.2, 0.387)	1.35 (0.37–4.95, 0.648)	1.2 (1.05–1.37, 0.006)

* Adjusted Odds Ratio (AOR) (for confounders: socio-demographics, hospital characteristics, the Charlson comorbidity index, and other cardiac risk factors, including tobacco abuse, hypertension, hyperlipidemia, diabetes, cannabis use, and obesity).

**Table 4:** Multivariate regression analysis of racial differences in the occurrence of in-hospital complications of SCD patients in the NIS (2016–2020).

Complications	White [22,710]	African American [981,759] AOR * [95% CI, *p*-Value]	Hispanic [52,176] AOR * [95% CI, *p*-Value]	Others [32,569] AOR * [95% CI, *p*-Value]
AKI	Reference	1.68 (1.46–1.94, 0.001)	1.31 (1.09–1.57, 0.003)	1.51 (1.25–1.83, 0.001)
Pain Crisis	Reference	3.61 (3.22–4.05, 0.001)	1.93 (1.69–2.21, 0.001)	2.11 (1.8–2.48, 0.001)
DVT	Reference	1.43 (1.09–1.87, 0.011)	1.01 (0.73–1.39, 0.949)	1.44 (1.01–2.03, 0.041)
Acute Chest Syndrome	Reference	1.8 (1.41–2.3, 0.001)	1.6 (1.22–2.11, 0.001)	1.56 (1.17–2.08, 0.002)
CVA	Reference	0.36 (0.29–0.44, 0.001)	0.4 (0.28–0.57, 0.001)	0.42 (0.28–0.64, 0.001)
PHTN	Reference	1.98 (1.61–2.44, 0.001)	1.69 (1.29–2.22, 0.001)	1.18 (0.89–1.56, 0.241)

* Adjusted Odds Ratio (AOR) (for confounders: socio-demographics, hospital characteristics, the Charlson comorbidity index, and other cardiac risk factors, including tobacco abuse, hypertension, hyperlipidemia, diabetes, cannabis use, and obesity). AKI: acute kidney injury; DVT: deep vein thrombosis; CVA: cerebrovascular accident; PHTN: pulmonary hypertension.

## Data Availability

Data for this study is derived from public domain resources. The findings of these studies are corroborated by data from the HCUP, which researchers can access through a standard application process and a signed data use agreement. The authors note that they did not receive any special access to the HCUP data used in this study (covering the years 2016–2020). They paid the necessary fee to obtain the NIS data, as specified in the HCUP Central Distributor’s fee schedule, which manages purchase requests and data use agreements (DUAs) for all users (https://www.hcup-us.ahrq.gov/tech_assist/centdist.jsp, accessed on 5 November 2024). Researchers wishing to access HCUP databases must complete the online HCUP DUA (https://www.hcup-us.ahrq.gov/tech_assist/dua.jsp, accessed on 5 November 2024) and agree to its terms. Additional details on how to apply for HCUP database purchases are available at ([https://www.distributor.hcup-us.ahrq.gov], accessed on 5 November 2024), and link for the NIS dataset can be found at www.hcup-us.ahrq.gov/nisoverview.jsp, accessed on 5 November 2024.

## Abbreviations

The following abbreviations are used in this manuscript:

SCD	Sickle Cell Disease
NIS	National Inpatient Sample
AOR	Adjusted Odds Ratio
CDC	Centers for Disease Control and Prevention
HCUP	Healthcare Cost and Utilization Project
ICD-10	International Classification of Disease-Tenth Edition-Clinical Modification
AKI	Acute Kidney Injury
DVT	Deep Vein Thrombosis
PHTN	Pulmonary Hypertension
CI	Confidence Interval
RBCs	Red Blood Cells
